# Severe Neurologic Disease in a Horse Caused by Tick-Borne Encephalitis Virus, Austria, 2021

**DOI:** 10.3390/v15102022

**Published:** 2023-09-29

**Authors:** Phebe de Heus, Zoltán Bagó, Pia Weidinger, Dilara Lale, Dagmar S. Trachsel, Sandra Revilla-Fernández, Kaspar Matiasek, Norbert Nowotny

**Affiliations:** 1 Clinical Unit of Equine Internal Medicine, University of Veterinary Medicine Vienna, Veterinärplatz 1, 1210 Vienna, Austria; phebe.de-heus@vetmeduni.ac.at (P.d.H.); dilara.lale@vetmeduni.ac.at (D.L.); dagmar.trachsel@vetmeduni.ac.at (D.S.T.); 2 Institute for Veterinary Disease Control Mödling, Austrian Agency for Health and Food Safety Ltd. (AGES), Robert Koch-Gasse 17, 2340 Mödling, Austria; zoltan.bago@ages.at (Z.B.); sandra.revilla-fernandez@ages.at (S.R.-F.); 3 Viral Zoonoses, Emerging and Vector-Borne Infections Group, Institute of Virology, University of Veterinary Medicine Vienna, Veterinärplatz 1, 1210 Vienna, Austria; pia.weidinger@vetmeduni.ac.at; 4 Section of Clinical & Comparative Neuropathology, Institute of Veterinary Pathology, Centre for Clinical Veterinary Medicine, Ludwig-Maximilians-Universität München, Feodor-Lynen Straße 23, 81377 Munich, Germany; kaspar.matiasek@neuropathologie.de; 5 Department of Basic Medical Sciences, College of Medicine, Mohammed Bin Rashid University of Medicine and Health Sciences, Dubai Healthcare City, Building 14, Dubai P.O. Box 505055, United Arab Emirates

**Keywords:** equine, tick-borne encephalitis, TBEV, flavivirus, neurologic disease, ataxia, vector-borne disease, leptomeningoencephalitis

## Abstract

As evidenced by sero-epidemiological studies, infections of horses with the tick-borne encephalitis virus (TBEV) occur frequently in TBEV-endemic areas. However, there are only very few reports of clinical cases. A possible underreporting may be due to a variety of diagnostic challenges. In this study, ELISA and neutralization tests were applied to serum samples. Brain tissue samples were investigated for the presence of nucleic acids of TBEV, *Equid alphaherpesvirus 1*, Borna disease virus 1, West Nile and Usutu viruses, rustrela virus, as well as Eastern, Western, and Venezuelan equine encephalitis viruses with RT-qPCR, RT-PCR, and qPCR, respectively. TBEV-specific amplification products were subjected to Sanger sequencing. In addition, a direct fluorescent antibody test for rabies was performed. Clinical and patho-histological findings are reported. Using specific RT-qPCR and RT-PCR assays, TBEV nucleic acids were demonstrated in brain tissue samples. Sequencing revealed the Western (formerly Central) European subtype of TBEV as the etiological agent. A high titer of TBEV-specific neutralizing antibodies was found in the serum. RNAscope in situ hybridization revealed TBEV RNA confined to neuronal cell bodies and processes. No other pathogens or nucleic acids thereof could be detected. Diagnostic procedures need to be carried out early after the onset of neurological signs to allow for a final etiological diagnosis of acute TBEV infections in horses.

## 1. Introduction

Tick-borne encephalitis (TBE) is a zoonotic infectious disease caused by the tick-borne encephalitis virus (TBEV; genus *Flavivirus*; family *Flaviviridae* [1,2]). Humans and several animal species are susceptible to TBEV infection, albeit clinical disease is mainly seen in humans and, less frequently, in certain animal species, such as dogs [3,4,5]. Although horses are often infected by TBEV in endemic areas [6,7], they rarely develop clinical disease. Transmission of TBEV occurs mainly through infected *Ixodes* spp. ticks. Non-vectorial transmission routes in humans are rare and have mainly been associated with the consumption of unpasteurized milk or raw milk products, such as cheese from acutely infected farm animals [8]. The infection in ruminants is usually asymptomatic; a rare fatal case was reported more than 20 years ago by our group [9]. Occasionally, infections were also demonstrated through the handling of infected material, such as blood, and through solid organ transplants [10].

TBE occurs predominantly in focal endemic geographic areas in Eurasia with climatic conditions favoring tick activity [11,12]. A significant rise in TBE cases was reported in Europe over the recent decades, including Austria [11,13]. In humans, Austria had the highest morbidity numbers in Europe before the introduction of a vaccine. Mass vaccination campaigns in Austria since 1981 contributed significantly to the dramatic decline in morbidity [11]. In Europe, no vaccines are licensed for veterinary use. Efficacy of vaccines was shown in goats, where viral shedding in milk could not be detected after immunization [14]. Several vaccine candidates intended for veterinary use are under development [15,16].

Sporadic reports of equine neurological cases associated with TBEV were published in 1981 from Switzerland [17] and in 1999 from Austria [18]. After almost two decades of silence, several cases from Austria (*n* = 1) [19], Germany (*n* = 1) [19], and Switzerland (*n* = 7) [20,21] were described. Seroprevalences in equines in Europe range from 0.8% in Northern Germany [22] to 37.5% in Lithuania [23]. In Catalonia (Spain), sporadic seropositivity was found without endemic foci [24]. Surveillance studies of equids residing in Austria revealed a consistently high prevalence of neutralizing antibodies over the years, from 26.1% in 2011 [6] to 15.5% in 2017 [7]. Also, neutralizing antibodies against TBEV were detected in donkeys [7].

In general, neurologic disease after TBEV infection is rare in horses, and diagnosis is often hampered by high antibody prevalence. As there are no pathognomonic signs, diagnosis often requires extensive testing, especially in areas where other flaviviruses such as the West Nile and Usutu viruses co-circulate—due to significant cross-reactivities between these viruses in most immunoassays [25,26,27]. Another diagnostic challenge is a consequence of the quick virus clearance, resulting in a negative reverse transcription (RT)-(q)PCR result on CNS specimens already a few days after the first clinical signs are observed. 

Diagnostic criteria to define TBE cases in equids have been defined by Fouché et al. and the European College of Equine Internal Medicine (ECEIM) [20,28]. The aim of this study was to describe the diagnostic process after inconclusive serological test results of a highly suspected acute equine TBE case, following the algorithms recommended by the ECEIM consensus statement. In addition, we aim to create awareness for clinical TBE cases in horses, which seem to have increased in recent years, but still remain underdiagnosed. 

## 2. Materials and Methods

The anamnesis, clinical findings, treatment, and outcome of the suspected acute equine TBE case were taken from the patient’s records. To rule out systemic and metabolic diseases that could trigger seizures, a complete blood count and biochemical analyses were performed. For the antemortem work-up of infectious diseases, several samples were taken, including serum and EDTA blood, in search of antibodies against various neuropathogenic agents.

### 2.1. Serological Tests—ELISA and Virus Neutralization (VN) Tests

Serum was collected in the morning after admission, about three days after the onset of clinical signs. The serum was sent to a commercial laboratory for the detection of antibodies to flaviviruses. For West Nile virus (WNV), IgG ID Screen West Nile Competition Multi-Species ELISA and ID Screen West Nile IgM Capture (both Innovative Diagnostics, Grabels, France) were performed. IgG and IgM antibodies against TBEV were analyzed using IMMUNOZYM FSME (TBE) IgG All Species ELISA (Progen, Heidelberg, Germany) and VetLine TBE/FSME Multi-Species IgM ELISA (NovaTec Immunodiagnostica, Dietzenbach, Germany), respectively. ELISA-reactive serum samples were further tested by virus neutralization (VN) assays against TBEV (isolate Salzburg/Milk/1432/2020) and WNV 1&2, developed at the national reference laboratory for equine encephalomyelitis (AGES, Mödling, Austria; unpublished, modified from the WOAH protocol [29]), to differentiate between these closely related flaviviruses present in Austria.

### 2.2. Investigation of the Liquor Cerebrospinalis

Cerebrospinal fluid (CSF) was collected through atlanto-occipital punction directly after euthanasia, about six days after the onset of neurologic signs. The CSF was used for standard in-house cytologic evaluation and protein concentration analysis. The presence of Borna disease virus-1 (BoDV-1) nucleic acid was analyzed with RT-PCR, as described previously [30].

### 2.3. Pathology and Histopathology

Following euthanasia of the horse, necropsy was performed. The head was sent to AGES to exclude notifiable infectious diseases. For histological examination, representative brain samples, the trigeminal ganglia, and the pituitary gland were embedded in paraffin wax; sections of 3–4 µm were cut and stained with haematoxylin and eosin.

### 2.4. Immunofluorescent and Molecular Tests Performed on Brain Tissue

The national reference laboratory performed a direct fluorescent antibody (DFA) test for rabies, as described by the World Organization for Animal Health (WOAH) [31], on the brain tissue of the horse. Furthermore, specific RT-qPCRs for WNV-1/2 [32], BoDV-1 [33], Eastern equine encephalitis virus (EEEV), Western equine encephalitis virus (WEEV), and Venezuelan equine encephalitis virus (VEEV) were also executed (following the standard operating procedures of the European Union Reference Laboratories, which are modified methods of [34,35]), as well as a universal pan-flavivirus RT-PCR [36] to detect other flaviviruses, such as Japanese encephalitis virus, TBEV, Usutu virus (USUV), WNV, and Zika virus.

In addition, eight small pieces of the brain tissue were sent to the Institute of Virology, University of Veterinary Medicine Vienna, for molecular analyses. Each sample was homogenized in 1 mL phosphate-buffered saline using two 2.8 mm ceramic beads (Bertin Technologies, Montigny-le-Bretonneux, France) and a TissueLyser II (QIAGEN, Hilden, Germany). Homogenates were then frozen to −80 °C for at least one hour, thawed, vortexed, and centrifuged for 2 min at 6200× *g*. Total nucleic acid extraction was performed with 140 µL of each supernatant using QIAamp Viral RNA Mini QIAcube Kit on a QIAcube device (both from QIAGEN), according to the manufacturer’s instructions. Extracts were screened with (RT)-(q)PCRs for the presence of the following viruses: TBEV, *Equid alphaherpesvirus 1* (EHV-1), BoDV-1, USUV, WNV, and rustrela virus (RusV). The respective primers and probes that were used are shown in Table 1. All PCRs included negative and positive controls.

At the Institute of Virology, University of Veterinary Medicine Vienna, RT-qPCRs were performed using qTOWER³ G (Analytik Jena, Jena, Germany) and Quantabio qScript XLT 1-Step RT-qPCR ToughMix (Quantabio, Beverly, MA, USA) with primers and probes at concentrations of 0.5 µM each, under the following conditions: 50 °C for 15 min, 95 °C for 2 min, and 45 cycles at 95 °C for 15 s, and 60 °C for 30 s. EHV-1 qPCR was performed using Rotor-Gene Q (QIAGEN) and Luna Universal Probe qPCR Master Mix (New England Biolabs, Ipswich, MA, USA) under the following conditions: 95 °C for 1 min and 45 cycles at 95 °C for 15 s, and 60 °C for 30 s.

Conventional TBEV RT-PCR was performed using QIAGEN OneStep RT-PCR Kit (QIAGEN) under the following conditions: 50 °C for 30 min, 95 °C for 15 min, 50 cycles of 94 °C for 30 s, 60 °C for 30 s, and 72 °C for 30 s, and a final elongation at 72 °C for 7 min. Products were examined by automatic gel electrophoresis on QIAxcel Advanced System (QIAGEN). Nucleotide sequences were obtained with Sanger sequencing using Mix2Seq Kits (Eurofins Genomics, Ebersberg, Germany), and identified with BLAST search (https://blast.ncbi.nlm.nih.gov/Blast.cgi, accessed on 27 February 2023).

### 2.5. RNAscope In Situ Hybridization

RNAscope probes detecting viral RNA were ordered and in situ hybridization was performed using RNAscope technology as described previously [19], with the RNAscope R (Red) 2.5 detection kit and a TBEV detection probe (cat. no. 575601; all from Advanced Cell Diagnostics, Bio-Techne, Minneapolis, MN, USA).

## 3. Results

### 3.1. Anamnesis and Clinical Findings

On the evening of 26 June 2021, a 16-year-old Friesian gelding was presented to the emergency service with a sudden onset of neurologic disease, manifesting as ataxia with a drift to the left, episodes of compulsive walking, presumed blindness, and head pressing for two days. Upon further questioning, the owner reported to have found the gelding wounded by a barbed wire fence several days prior. Further, the owner noticed inappetence, decreased fecal production, and a fever of 38.8 °C. A veterinarian that was consulted in the field had performed a rectal examination and treated the horse with paraffin oil per nasogastric tube and flunixin meglumine via intravenous injection. The horse was vaccinated against tetanus according to the owner, but the equid passport was not available to verify this or other vaccinations. The horse was purchased four months before and pastured in Lower Austria with four other horses that did not have any clinical signs. The owner mentioned an abundant tick infestation of the patient.

Upon initial presentation to the clinic, the gelding was lethargic. The horse was able to be examined in the stocks and stood quietly. Multiple superficial abrasions and scars covered the entire body. The heart rate was increased (60 beats/minute) as well as the breathing rate (18 breaths/min) and the rectal temperature (38.7 °C). The mucosal membranes were dry and moderately red. A rectal examination revealed a ten-centimeter-long white worm suspected to be *Oxyuris equi,* but was otherwise unremarkable. Ultrasonography of the thorax and abdomen was also unremarkable apart from mildly increased peritoneal fluid. The nasogastric tube yielded physiological gastric contents. Abnormal findings in the neurological exam were the absence of the left menace response, delayed pupillary light reflexes in both eyes, a stiff gait, compulsive walking with a drift to the left, and mild hypermetria. Chewing gum fits and bruxism were also present among the clinical signs. On the way to the box, a sudden deterioration manifested in episodic high-grade ataxia, muzzle tremors, and head turn to the left, lasting for less than a minute. Further, hypersensitivity to sounds, hyperesthesia, and left facial nerve paresis were noted the next day. The anatomic location of the lesion was suspected to be in the cerebrum and brain stem.

### 3.2. Treatment and Outcome

Treatment involved initial administration of intravenous crystalloid isotonic fluids (bolus and a maintenance rate afterwards) to correct fluid deficits. Lactulose (2 mL/kg, p.o.) was given per nasogastric tube as a treatment and prophylaxis was given against potential hyperammonemia before blood ammonia was known. The skin abrasions were cleaned and disinfected with a wound irrigation solution (octenilin). Symptomatic treatment was started with nonsteroidal anti-inflammatory drugs (flunixin meglumine, 1.1 mg/kg, i.v.) and glucocorticoids (dexamethasone, 0.1 mg/kg, i.v.) against presumptive encephalitis. In addition, the gelding received a gastric protectant (omeprazole, 1 mg/kg, p.o.). Acute seizure activity was treated with diazepam (0.01 mg/kg) as needed. After four days of treatment, no improvement of the clinical signs was noted. Short focal and generalized seizures continued (Supplementary Video S1). This resulted in self-harm, causing considerable soft tissue trauma around the left eye. Phenobarbital, for the purpose of reducing seizures, was already discussed with the owner before the trauma occurred. Further anti-epileptic treatment was, however, declined for financial reasons. Euthanasia was elected four days after admission.

### 3.3. Complete Blood Count and Biochemistry

Blood was collected at admission and analyzed in the emergency laboratory for a complete blood count (Procyte, IDEXX, Hoofddorp, The Netherlands) and selected biochemistry parameters (Catalyst One, IDEXX) (Table 2). About 18 h after admission, three days after the presumed onset of neurologic signs, blood was collected for analysis at the central laboratory. Complete blood count (Advia 2120i, Siemens, Vienna, Austria) and biochemistry (Cobas c501, Roche, Vienna, Austria) at the central laboratory revealed a mild monocytosis [563 cells/μL; reference range (rr) < 500 cells/μL], decreased urea (11.2 mg/dL; rr 20–40 mg/dL), triglyceridemia (109 mg/dL; rr < 50 mg/dL), increased creatine kinase (1101 U/L; rr < 200 U/L), increased serum amyloid A (755 mg/L; rr < 10), and increased lactate (2.8 mmol/L; rr < 1 mmol/L). Creatinine, total protein, albumin, glutamate dehydrogenase (GLDH), gamma-glutamyl transferase (GGT), ammonia, and electrolytes (sodium, potassium and chloride) were within normal limits (Cobas b124, Roche).

### 3.4. Results of the Virological Investigations of Whole Blood

EDTA blood was negative for WNV and TBEV nucleic acids. 

### 3.5. Serology

The WNV IgG competition reacted positive, while the WNV IgM ELISA reacted negative with values of 13.75% (rr > 50% negative) and 2.35% (rr < 35% negative), respectively. IgG antibodies against TBEV were found positive (> 500 U/mL; rr < 63 U/mL negative), while the anti-TBEV IgM antibodies showed a borderline result with 29.17 relative units (RU; cut-offs for negative and positive interpretation were referenced at <25 RU and >30 RU, respectively) (Table 3). Confirmatory testing for the presence of neutralizing antibodies in the horse’s serum using a VN assay revealed an estimated endpoint neutralization titer of 1:226, as calculated using the Spearman and Kärber method [41,42]. Although no cut-off for a positive response is defined, we considered this as distinct evidence for the presence of neutralizing antibodies against TBEV, as a positive control serum, derived from the European Union reference laboratory for equine diseases (ANSES, Maisons-Alfort, France), achieved significantly lower titers (1:20). No unspecific reactions, confirmed by negative controls, were observed. To exclude cross-reactions, VN assays against WNV lineages 1 and 2, performed according to the standardized WOAH protocol [29], were conducted and yielded negative results.

### 3.6. Cerebrospinal Fluid

The CSF showed mild xanthochromia and mild blood contamination with a cell count of 32 cells/μL. Moderate lymphocytic pleocytosis was noted with 97% lymphocytes, 2% macrophages, and 1% neutrophils. Total protein (102 mg/dL; rr < 48 mg/dL) and glucose (89 mg/dL; rr: 40–70 mg/dL) were increased. BoDV-1 nucleic acid was not detected in the CSF.

### 3.7. Pathology and Histopathology

Necropsy revealed the injury above the eye to be purely soft tissue trauma including a hematoma. The internal organs showed no relevant pathomorphological alterations.

**Figure 1:**
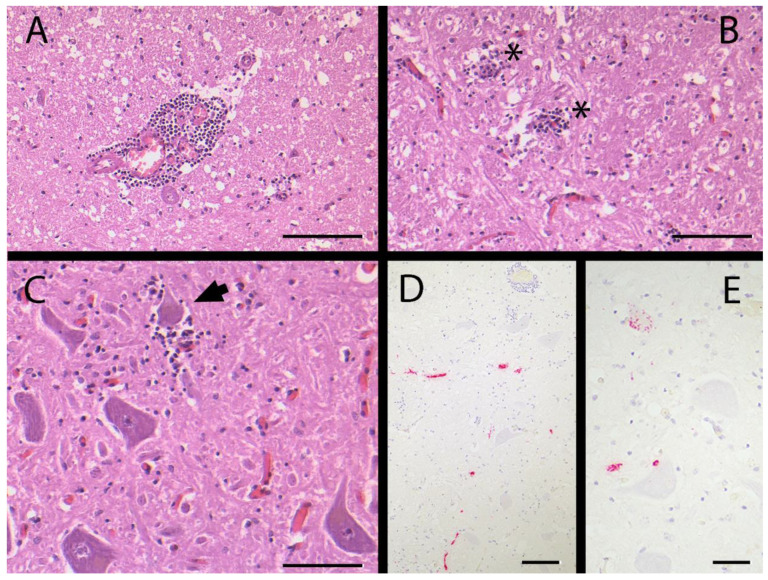
TBEV-associated lesions in the affected horse. (**A**) Moderate lymphomonocytic perivascular infiltrate in the center of the picture, microphoto, HE, bar = 180 µm. (**B**) Slight glial nodules in the neuroparenchyma (asterisks), microphoto, HE, bar = 180 µm. (**C**) Early stage neuronophagy with microgliosis (arrow), microphoto, HE, bar = 100 µm. (**D**,**E**) RNAscope in situ hybridization for TBEV RNA. Red positive signals are seen in neuronal processes (**D**) and neurons (**E**), microphoto, bar = 180 µm (**D**) and 50 µm (**E**).

The head of the animal was sent to AGES to exclude notifiable infectious diseases. Standard protocols were followed. Histopathology of the brain revealed moderately acute non-suppurative leptomeningoencephalitis characterized by multifocal lymphomonocytic perivascular infiltrates, and some glial nodules occasionally accompanied with neuronophagy (Figure 1A–C). These angiocentric and neuronocentric lesions were predominantly associated with the brain stem. The trigeminal ganglion displayed mild focal lymphocytic ganglionitis. As an additional finding, a well-differentiated microadenoma of the pars intermedia of the hypophysis was detected.

### 3.8. Results of the DFA Test for Rabies and Molecular Tests for the Presence of Nucleic Acids of Neuropathogenic Viruses in Brain Tissue

The direct fluorescent antibody (DFA) test on brain tissue for rabies virus reacted negatively. Specific RT-qPCRs for WNV-1/2, BoDV-1, EEEV, WEEV, and VEEV on brain tissue were also negative. The universal pan-flavivirus conventional RT-PCR did not show any visible PCR bands.

All (RT)-(q)PCRs performed on brain tissue that was sent to the Institute of Virology, University of Veterinary Medicine Vienna, were negative, except for TBEV. Seven out of eight brain samples were positive in the TBEV RT-qPCR (C_t_ values 31.1–38.6), and from three samples specific bands appeared in the conventional TBEV RT-PCR. Sanger sequencing resulted in two identical sequences from two specimens, most closely related to strains isolated in Finland in 2013 and in Germany in 2011 (99.1%), as well as in Slovenia in 2008 and in former Czechoslovakia in 1953 (98.6%), revealing the Western (or Central) European subtype of TBEV.

### 3.9. RNAscope In Situ Hybridization for TBEV RNA

RNAscope-positive signals were confined to neuronal cell bodies and processes (Figure 1D,E), even distant from other tissue changes such as lymphomonocytic perivascular infiltrates.

## 4. Discussion

Horses are susceptible to TBEV infections, as evidenced by high antibody prevalence in TBE endemic areas [6,7]. They may even be considered excellent sentinel animals for the presence of TBEV and other flaviviruses in certain geographic regions [43]. Cases of clinical TBE in horses, however, are rare or underdiagnosed—due to several diagnostic limitations and challenges. The diagnosis of presumable cases reported in the recent decades were mainly based on clinical signs, serology, and sometimes histopathology. Unequivocal etiologic diagnosis was often not achieved [17,18]. Recent reports described clinical signs in more detail and based the diagnosis on the presence of TBEV-specific IgM antibodies and/or the increase of TBEV-specific (neutralizing) antibodies in paired serum samples [20,21]. Complete blood count and biochemistry is non-specific but, nonetheless, important to rule other differential diagnoses. Our case had leukocytes within normal limits with initial neutrophilia and monocytosis, in contrast to six acute cases from Switzerland [21] which all showed leukocytosis, and another case which displayed neutrophilia and lymphopenia [20]. These changes in white blood cell count could represent different phases of disease. Monocytosis is usually seen with resolving or long-lasting inflammation. Serology is hampered by cross-reactions with antibodies to other flaviviruses, such as WNV [25,26,27]. Our case illustrates this cross-reaction in ELISAs, as both IgG tests for WNV and TBEV reacted positive. The viral neutralization clarified this unequivocally as a cross-reaction, as the WNV neutralization test was negative in comparison to the positive TBEV viral neutralization test. Consequently, reactivity in ELISAs has to be confirmed by the much more specific neutralization assay, which, however, requires a biosafety level 3 (BSL-3) laboratory. Regarding the molecular detection of TBEV, the universal pan-flavivirus RT-PCR [36] is often less sensitive to detect lower viral loads, as we experienced in the case described here as well. Also, due to different distribution patterns of the TBEV RNA and antigen, more brain localizations should be tested by molecular analyses separately and should not be pooled, as we did initially.

Horses infected by TBEV can present various clinical signs depending on the affected location of the nervous system. In humans, neurological clinical signs can be present as meningitis, meningoencephalitis, and meningoencephalomyelitis. The signs of meningitis are fever, exhaustion, and photophobia, which might proceed to meningoencephalitis with alterations in consciousness and neurological deficits. Flaccid paralysis occurs with further progress to meningoencephalomyelitis [2]. So far, the reported clinical signs detected in horses are anorexia, altered mentation, ataxia, epileptiform seizures with or without sudden cramps, paralysis, and reduced general condition [44]. Conze et al. [19] reported two horses with a fatal outcome, where the first one was presented with grade 4/5 ataxia, difficulty to stop moving, altered mentation, and bilateral lack of menace response, whereas the second horse only was presented with grade 4/5 ataxia. The six cases reported by Magouras et al. [21], where five horses were discharged, were presented with abnormalities in mentation (in four horses) and cranial nerves (in two horses), ataxia from grade 2/5 to 4/5, hyperesthesia (in five horses), and tremors (in two horses), as well as twitching of nostrils, photophobia, stringhalt-like gait, and muscle spasm in some horses. Fouché et al. [20] reported a case with normal mentation, unilateral facial nerve paralysis, fine muscle fasciculations, and tremors around the face that was discharged after treatment. The reports above reveal the variable clinical presentation in infected horses, indicating that further research is needed to detect associations of clinical signs with the outcome, and to differentiate other neurological diseases. The case described in this report is relatively unique, as it displayed seizure activity, which has rarely been reported (Supplementary Video S1).

Thus, how to proceed?

TBEV in horses should be considered as differential diagnosis when horses are presented with neurologic signs and have been in TBEV endemic areas roughly between May and October. Tick infestation supports a tentative diagnosis. All three elements apply to the case described in this paper. Detailed diagnostic criteria for TBEV in equids have been defined by Fouché et al. and the ECEIM [20,28].

This report describes the diagnostic processes of a case of acute equine TBE following initial inconclusive serological testing. We largely followed the algorithms recommended by the ECEIM consensus statement. A tentative diagnosis of TBE was made based on the antemortem serology and was confirmed with postmortem analyses of the brain tissue using TBEV-specific RT-qPCR, conventional RT-PCR with subsequent sequencing of the amplicons, and RNAscope in situ hybridization technology. A clear-cut etiological diagnosis can be achieved with a combination of these techniques. Most of these techniques (except conventional RT-PCR and subsequent sequencing) were also recently applied successfully to Austrian and German cases of equine TBE [19]. However, due to the quick clearance of TBEV from the infected tissue, the suggested etiological diagnosis has to be initiated as quick as possible. 

Metabolic differential diagnoses such as hepato-encephalopathy were largely excluded due to unremarkable biochemistry (including ammonia) and electrolytes. Bacterial meningitis was also considered among the differential diagnoses, but antemortem CSF puncture for further diagnosis or initial antimicrobial treatment was declined by the owner. Differential diagnoses must also cover other viral pathogens potentially leading to CNS diseases in horses in the respective area. This includes (in Austria) testing for EHV-1 [45], BoDV-1 [30,46,47], WNV [48], USUV, and possibly the recently identified RusV [40,49,50], as well as rabies virus, although wildlife rabies is no longer present in Central Europe. Even though highly unlikely, we also screened for the American EEEV, WEEV, and VEEV, according to the Animal Health Law, and for the Japanese Encephalitis Virus. However, no nucleic acids of any of the above viruses were found in this case. 

As expected, partial TBEV nucleotide sequences derived from two brain samples of the described case revealed the Western European subtype of TBEV, also called the Central European subtype [1], as the causative agent, genetically closely related to virus strains from Finland, Germany, Slovenia, and former Czechoslovakia. Viral subtypes have an effect on clinical progression in humans, whereby the Far Eastern subtype shows higher mortality than the European and Siberian subtypes. Also, the European subtype causes a biphasic disease course, whereas the Siberian and Far Eastern subtypes mostly lead to a monophasic course of disease [2]. Thus far, in equids, there is no further information regarding the pathogenicity of different variants, which requires further investigation. 

## 5. Conclusions

This report describes a rare case of TBEV in a horse presented with neurologic clinical signs. Thanks to advanced technologies, it is meanwhile possible to unequivocally establish the diagnosis of TBEV infection. We report the diagnostic procedures which led to the diagnosis of TBEV infection in a horse, and demonstrated the exclusion of other potential neurotropic pathogens. This report might encourage other clinicians to consider TBEV infection as differential diagnosis in similar cases, and to consider and combine further diagnostic approaches since TBE in horses is still an underdiagnosed disease.

## Figures and Tables

**Table 1:** PCR primers and probes used for the detection of various viruses.

Virus (Family)	Primer/Probe Name	Sequence (5′-3′)	Amplicon Length	Reference
TBEV (*Flaviviridae*)	TBE1-F TBE1-R TBE1-P(WT)	GGGCGGTTCTTGTTCTCC ACACATCACCTCCTTGTCAGACT TGAGCCACCATCACCCAGACACA	67 bp	RT-qPCR; [37]
	TBE1-6895F TBE4-7128R	GGACTGGTTGCAGCCAATGA AGATGCCACGGCACTGTTGA	233 bp	RT-PCR; modified from [9]
EHV-1 (*Herpesviridae*)	EHV1-29F EHV1-82R EHV1-P	ATCTGGCCGGGCTTCAAC GGTCACCCACCTCGAACGT ATCCGTCRACTACTCG	53 bp	qPCR; modified from [38]
BoDV-1 (*Bornaviridae*)	BoDV1-F(p24) BoDV1-R(p24) BoDV1-P(p24)	TCCCTGGAGGACGAAGAAGAT CTTCCGTGGYCTTGGTGACC CCAGACACTACGACGGGAACGA	69 bp	RT-qPCR; modified from [33]
USUV (*Flaviviridae*)	USUV-9721F USUV-9795R USUV-9746P	GCCAATGCCCTGCACTTT TCCCGAGGAGGGTTTCCA CGATGTCCAAGGTCAGAAAAGACGTGC	74 bp	RT-qPCR; [39]
WNV (*Flaviviridae*)	WNV-8F WNV-118R WNV-67P	CGCCTGTGTGAGCTGACAAA GCCCTCCTGGTTTCTTAGACATC TGCGAGCTGTTTCTTAGCACGA	110 bp	RT-qPCR; in-house method for WNV lineages 1 and 2 [32]
RusV (*Matonaviridae*)	RusV-234F RusV-323R RusV-256P	CCCCGTGTTCCTAGGCAC TCGCCCCATTCWACCCAATT GTGAGCGACCACCCAGCACTCCA	89 bp	RT-qPCR; [40]
Flaviviruses (*Flaviviridae*)	MAMD cFD2 FS 778	AACATGATGGGRAARAGRGARAA GTGTCCCAGCCGGCGGTGTCATCAGC AARGGHAGYMCDGCHATHTGG	252 bp	RT-PCR; [36]
EEEV *(*Tog*a*viridae*)*	EEE Fwd EEE Rev EEE probe	ACACCGCACCCTGATTTTACA CTTCCAAGTGACCTGGTCGTC TGCACCCGGACCATCCGACCT	69 bp	RT-qPCR; [35]
WEEV *(Togaviridae)*	WEE Fwd WEE Rev WEE probe	CTGAAAGTCGGCCTGCGTAT CGCCATTGACGAACGTATCC ATACGGCAATACCACCGCGCACC	67 bp	RT-qPCR; [35]
VEEV *(Togaviridae)*	VEE Fwd VEE Rev VEE probe	TCCATGCTAATGCYAGAGCGTTTTCGCA TGGCGCACTTCCAATGTCHAGGAT TGATCGARACGGAGGTRGAMCCATCC	98 bp	RT-qPCR; [34]

**Table 2:** Hematology and biochemistry results at admission and one day after admission.

Parameter	Emergency Admission * (2 Days after Onset of Signs)	Reference Range * Emergency Laboratory	1 Day after Admission** (3 Days after Onset of Signs)	Reference Range ** Central Laboratory
Leukocytes (/µL)	10,240	4900–11,100	9230	5000–10,000
Neutrophils (/µL)	***7220***	2500–6900	6691	3000–7000
Lymphocytes (/µL)	2320	1000–4500	1661	1000–4500
Monocytes (/µL)	***660***	200–600	***563***	<500
Eosinophils (/µL)	20	0–800	221	<500
Basophils (/µL)	20	0–100	92	<200
LUS (/µL)	-	-	9	<100
Urea (mg/dL)	-	-	***11.2***	20–40
BUN (mg/dL)	***9***	10–25	-	-
Creatinine (mg/dL)	1.3	0.8–2.2	0.9	<2
Total protein (g/dL)	-	-	7.1	5.5–7.5
Albumin (g/dL)	2.9	1.9–3.2	3.2	2.4–4.5
GLDH (U/L)	-	-	3	<13
GGT (U/L)	-	-	13	<30
Triglycerides (mg/dL)	-	-	***109***	<50
Creatine kinase (U/L)	-	-	***1101***	<200
Ammonia (µmol/L)	-	-	below detection	<7
Serum amyloid A (mg/L)	-	-	***755***	<10
Sodium (mmol/L)	136	136–145	-	-
Potassium (mmol/L)	3.68	3.5–5.1	-	-
Chloride (mmol/L)	100	98–107	-	-
Ionized calcium (mmol/L)	***1.45***	1.15–1.33	-	-
Lactate (mmol/L)	***2.8***	1.0–1.8	***2.8***	<1

LUS = large unstained cells; BUN = blood urea nitrogen; GLDH = glutamate dehydrogenase; GGT = gamma-glutamyl transferase. * At admission, blood was analyzed at the emergency laboratory; ** one day after admission, blood was analyzed at the central laboratory using different analyzers. Therefore, reference ranges differ between the laboratories. Values in bold italics are outside the respective reference range.

**Table 3:** Serological results—ELISA and virus neutralization (VN) tests.

Antibody	Test	Company	Result	Interpretation
WNV IgG	ID Screen West Nile Competition Multi-Species IgG ELISA	Innovative Diagnostics	13.75% S/P	positive
WNV IgM	ID Screen West Nile IgM Capture ELISA	Innovative Diagnostics	2.35% S/P	negative
TBEV IgG	IMMUNOZYM FSME (TBE) IgG All Species ELISA	Progen	>500 U/mL	positive
TBEV IgM	VetLine TBE/FSME Multi-Species IgM ELISA	NovaTec Immunodiagnostica	29.17 RU	borderline
TBEV	VN (isolate Salzburg/Milk/1432/2020)	AGES in-house method	titer 1:226	positive
WNV 1 and 2	VN (2.2 neutralization protocol)	WOAH	titer < 1:5 (both)	negative

## Data Availability

The data presented in this study are available on request from the corresponding author. The data are not publicly available due to privacy reasons.

## Supplementary Materials

The following supporting information can be downloaded at: https://www.mdpi.com/article/10.3390/v15102022/s1, Video S1: de Heus et al., clinical signs of tick-borne encephalitis in a horse. Despite four days of treatment, short focal and generalized seizures continued.
